# Exploring the first-time transition to parenthood in mainland China: a qualitative study on the experiences of fathers and mothers using the transition shock model

**DOI:** 10.3389/fpsyg.2024.1249211

**Published:** 2024-06-07

**Authors:** Xi Lang, Tieying Zeng, Sha Ni, Lingjun Jiang, Pan Qian, Meiliyang Wu

**Affiliations:** ^1^Department of Nursing, Tongji Hospital, Tongji Medical College, Huazhong University of Science and Technology, Wuhan, China; ^2^School of Nursing, Tongji Medical College, Huazhong University of Science and Technology, Wuhan, China

**Keywords:** transition to parenthood, fatherhood, motherhood, qualitative study, transition shock, dyadic analysis

## Abstract

**Background:**

The transition to parenthood, which is influenced a lot by local parenting culture, is a dramatic stress for both men and women. Chinese social and cultural contexts form specific parental culture, shaping the unique experience of transition to parenthood. However, the understanding of the transition to parenthood in mainland China is limited. Additionally, few qualitative studies explored the transition to parenthood from both dyadic perspectives.

**Aim:**

To explore the first-time transition to parenthood experience among mothers and fathers in mainland China during pregnancy, and compare the similarities and differences between their experiences in this transition period.

**Methods:**

A descriptive qualitative study was conducted with 36 parents, including 18 primiparous women and their husbands. Data were analyzed by directed content analysis guided by the Transition Shock Model. The interview texts were first analyzed at individual levels and subsequently at the couple level to identify dyadic themes.

**Results:**

Five themes and thirteen sub-themes emerged from the data analysis, including role integration, health risk, dilemma of preparation, protective isolation, and multi-dimensional expectation. Unexpectedly, the experiences and perspectives of mothers and fathers regarding the transition to parenthood were found to be similar, with the exception of the sub-theme extra-care requirement.

**Conclusion:**

The findings shed light on the complex emotional journey and expectations of parents, as well as the challenges they face in terms of physical well-being, limited coping resources, and restricted social connections. Notably, fathers in China often shared the stress of the whole process during the transition period alongside mothers but often lacked accessible avenues for seeking and receiving support. These findings underscore the importance of actively involving fathers as a key support population in perinatal care, as well as the need for comprehensive support systems and tailored interventions to enhance the well-being and adaptation of parents.

## 1 Introduction

The transition to parenthood refers to the shift from the established and familiar roles of partners to the new roles of parents. However, rather than being filled with joy and confidence in the new parental role, many parents were struggling with discomfort, feelings of uncertainty, stress, anxiety, and depression during this period (Deave et al., 2008; Figueiredo and Conde, 2011; Dabb et al., 2023).

Extensive research has shown that this period significantly impacts parents’ health and well-being, parent–child relationship, and infant development (Fenwick et al., 2012; Philpott et al., 2017). A longitudinal study conducted from pregnancy to the postnatal period observed that parents’ well-being in couple relationships decreased significantly during the pregnancy (Moreno-Rosset et al., 2016). Two meta-analyses have indicated a high prevalence of perinatal anxiety among parents, demonstrating that 16.1–18.8% of mothers and 8.1–13.9% of fathers experience varying levels of anxiety during the perinatal period (Yang et al., 2020; Leiferman et al., 2021). What’s worse, maternal perinatal anxiety was found to increase the risks of lower birth weight and child developmental delay (Estinfort et al., 2022). Importantly, it is also crucial to raise awareness that parents who experience poor adaptation to parental roles are at a higher risk of engaging in child abuse (Gonzalez and Rodriguez, 2023), which can have detrimental effects on children’s psychological and physical well-being.

Many previous studies have explored the transition experiences from the perspectives of either mothers or fathers. Qualitative studies have identified various challenges faced by parents, which are often regarded as painful and negative events (Darvill et al., 2010; Baldwin et al., 2018; Mahaffey et al., 2021). Mothers have expressed complaints that enduring physical discomfort, concerns about their baby’s health, changes on body shape, heightened maternal role expectations, and dietary restrictions (Bianchi et al., 2016; Ladekarl et al., 2022). Fathers, on the other hand, have reported multiple concerns related to pregnancy and childbirth about the pregnancy news, their partners, the baby, care-giving skills, and financial matters (Baldwin et al., 2018; Dabb et al., 2023). Both mothers and fathers reported undergoing an adjustment in their priorities, values, and responsibilities as they shifted their focus from themselves to their children (Draper, 2003; Chang et al., 2010), accompanied by a sense of losing freedom. Furthermore, the weighty responsibilities of caring for and raising a baby led to feelings of overburden and a sense of incompetent (Soltani et al., 2017; Ladekarl et al., 2022). Notably, first-time parents are more likely to experience doubts and uncertainties regarding their ability to be qualified parents, due to lacking prior coping experiences (Lévesque et al., 2020; Ladekarl et al., 2022).

Existing research on the transition to parenthood has primarily examined the experiences of either mothers or fathers, overlooking the understanding of both partners’ perspectives (Lévesque et al., 2020; Collaco et al., 2021). Consequently, the similarities and differences in transition experiences between fathers and mothers remain uncertain. Furthermore, the thorough investigation of all challenges associated with the transition to parenthood and the potential positive effects of these events on the transition process are still unclear, particularly in the absence of theoretical guidance. This highlights the need for a well-established theory to steer our investigation towards acquiring a deeper comprehension of the shared and distinctive experiences of both mothers and fathers.

Meanwhile, the cultural context has been revealed as a significant impact on shaping social expectations and stereotypes related to parental roles, which can create additional stress for parents. For example, mothers in Japan and South Korea face severe social judgment to meet family and social expectations to devote themselves fully to the care of their children and sacrifice their own careers (Chang et al., 2010; Song et al., 2016; Katou et al., 2022), while studies conducted in western countries seldom mentioned this kind of stress.

Similar to other Asian countries, China also has the preference for mothers to take on the primary responsibility for child-rearing. However, China possesses a distinctive and blended social-cultural context that inherits traditional parental values alongside modern parental concepts. In China, mothers are not limited to the home but rather strive to balance their maternal roles with other societal roles, which may result in special transition experiences (Pan and Sun, 2022). Moreover, as the demand for fathers’ engagement increases, parenting has become a shared responsibility and challenge for both fathers and mothers in China.

To address these gaps, we conducted a qualitative study from a dyadic perspective, enabling us to delve deeper and obtain a more comprehensive understanding of the first-time transition to parenthood of both mothers and fathers in China. In order to guide our investigation, we adopted the Transition Shock Model (TSM) as the theoretical framework in this study. The TSM was first proposed by Duchscher in 2009, providing a framework to conceptualize and categorized the changes and challenges in the transition process. The intensity and duration of transition shock are depended on how apparent the contrast is between the different required expectations of the former role and the unfamiliar role. Although the Transition Shock Model was not initially developed for expectant parents’ transition experiences, it has been expanded to other role-shifting experiences (Sun and Xu, 2019; Rees et al., 2021). This suggested we could explore parents’ transition experience through these four categories to advance our understanding (Gwadz et al., 2018). The TSM divides role transition experience into four aspects: roles, responsibilities, relationships, and knowledge (Duchscher, 2009; Duchscher and Windey, 2018), which provided a structured approach to understanding the challenges encountered by parents during this critical period, and allowed us to capture a more nuanced understanding of the experiences and perspectives of Chinese parents as they navigate the transition to parenthood. The theory application could enhance our sensitivity in identifying relevant concepts and themes associated with the transition to parenthood experienced in China (Graneheim and Lundman, 2004; Macfarlane and O'Reilly-de Brun, 2012).

## 2 Materials and methods

### 2.1 Study design

Α descriptive qualitative design was utilized, employing in-depth interviews to gather rich and detailed data (Colorafi and Evans, 2016). To ensure a more open and candid discussion, separate interviews were conducted with each member of the couple rather than conducting joint interviews. This decision was based on the consideration that when partners are present, parents may find it challenging to openly express negative experiences or concerns about their partner (Leichtentritt and Weinberg Kurnik, 2020).

### 2.2 Ethical consideration

This study received ethical approval (NO.TJ-IRB20220705) from the Ethics Committees of Tongji Hospital of Tongji medical college of Huazhong University of Science and Technology. All the study investigators had no prior relationships with participants. The principal investigator provided a detailed explanation of the research objectives and methods to each participant. Informed consent was obtained, and participants were informed of their right to withdraw from the study at any point. Prior to each interview, the investigators assured the participants that their personal identifiers would not be used in any publications or reports. If participants exhibited significant emotional distress during specific questions, the interview was paused, and appropriate support and comfort were provided. Additionally, participants were not compelled to answer any questions they were uncomfortable with, and the investigators respected their decision and did not insist on obtaining a response.

### 2.3 Participants

The participants for this study were recruited from the Obstetrics outpatient department of Tongji Hospital, Tongji Medical College, Huazhong University of Science and Technology in China. Purposeful sampling was employed to ensure diversity among the participants. The inclusion criteria for the pregnant couples were as follows: (1) legally married couples, (2) primiparous couples, and (3) signed informed consent. Due to the focus on exploring the interaction between the couples, data from both the mother and father were required. Recruitment of couples continued until data saturation was reached, meaning that no new themes or insights emerged from the interviews. In total, 18 couples were recruited to participate in the study, including 18 primiparous and their husbands. The median age of mothers was 31.5 years (Inter quartile range, IQR: 28.25–33 years). The median age of fathers was 32.5 years (IQR: 29–34 years). Table 1 shows more detailed information about expectant parents.

**Table 1:** Demographic characteristics of mothers(*N* = 18) and fathers (*N* = 18).

Demographic characteristics	Mother		Father	
	*N*	%	*N*	%
Residence
Urban	15	83.3	14	77.8
Rural	3	16.7	4	22.2
Educational level
Undergraduate	16	88.9	15	83.3
Middle school	1	5.6	2	11.1
Primary school	1	5.6	1	5.6
Work status
Working	11	61.1	17	94.4
Leave of absence	5	27.8	0	0
Unemployed	2	11.1	1	5.6
Monthly per capita household income (RMB)
<10,000	2	11.1		
10,000–20,000	15	83.3		
>20,000	1	5.6		
Pregnancy trimester				
First trimester	6	16.2		
Second trimester	8	21.6		
Third trimester	4	10.8		
Planned pregnancy				
Yes	16	88.9		
No	2	11.1		
Miscarriage history				
Yes	5	27.8		
No	13	72.2		

### 2.4 Data collection

The interviews for this study were conducted by the investigator between July and November 2022. The interviewers usually interviewed mothers first, as they could provide more detailed information about the challenges experienced during pregnancy. Each interview lasted between 20 and 60 min, depending on the participant’s willingness to share their experiences. In addition to the qualitative data collected during the interviews, social-demographic information was also collected from each participant. This information included age, place of residence, pregnancy trimester, work status, educational level, household income, whether the pregnancy was planned, and any history of miscarriage.

A semi-structured interview approach was employed in this study, utilizing an interview question guide that was developed based on the Transition Shock Model (TSM), a comprehensive literature review, and internal discussions within the research team. To ensure the effectiveness of the interview guide, a pilot study was conducted with 3 couples to test its appropriateness and clarity.

**Figure 1:**
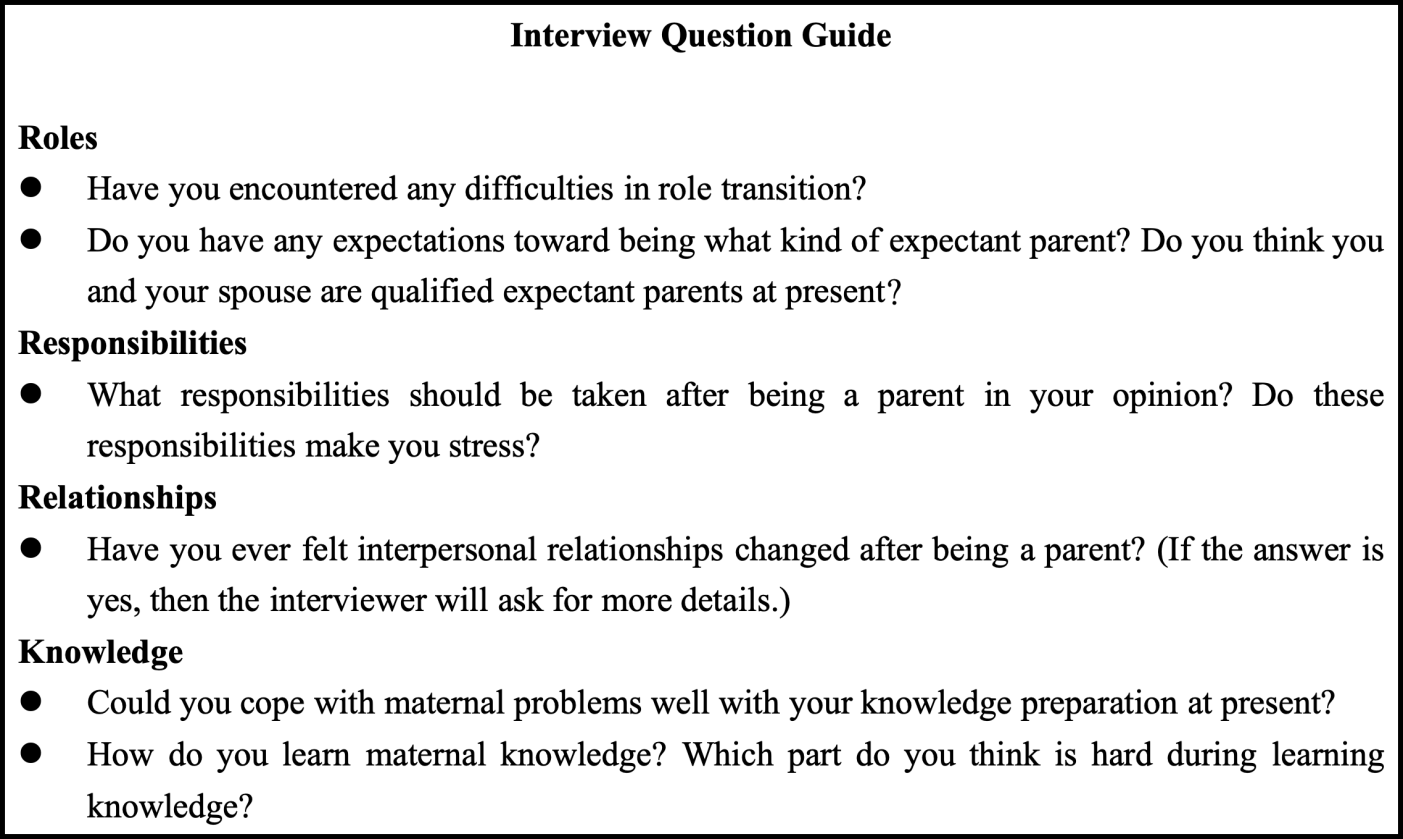
Interview question guide.

During the actual interviews, the interviewer began with a broad question, such as “What changes or challenges did you experience after becoming parents?” This open-ended question allowed participants to freely express their thoughts and experiences. If participants indicated that they were experiencing stress or strain while answering the question, the interviewer would gently inquire if they were willing to provide more detailed information to gain a deeper understanding of their experiences. The interview question guide is displayed in Figure 1.

The termination of data collection depended on information saturation. All interviews were audio-recorded and transcribed word by word. The non-verbal behaviors were noted to avoid losing information.

### 2.5 Data analysis

All audio recordings and interview notes were promptly transcribed into written texts by the researchers, who listened to the recordings repeatedly to ensure accuracy. To protect the participants’ privacy, each participant was assigned a unique code name, consisting of an identifier and a numeric suffix. Specifically, “M” represented mothers, and “F” represented fathers, with each couple sharing the same numeric suffix.

The data analysis process involved the application of directed content analysis and dyadic analysis methodologies, drawing from established references in the field (Colorafi and Evans, 2016; Collaco et al., 2021). The TSM, encompassing the dimensions of Roles, Relationships, Responsibilities, and Knowledge, served as the conceptual features for coding the data. After general codes and themes were formed at individuals’ level, the couples were matched according to their same numeric suffix. Dyadic analysis was used to analysis dyadic data to get the couples’ level of experiences instead of individuals’ level of experience. To ensure rigor in the analysis, two analysts independently examined the transcripts and coded the data, allowing for inter coder agreement and discussion to ensure consensus. The analysis method is shown in detail in Table 2. Examples of analytic process are given in Table 3.

**Table 2:** The methods of data analysis.

Analysis Stage	
Stage1: Transcription	All audio recordings and interview notes were transcribed into texts within 24 h after the interviews.
Stage2: Familiarization	Familiarization with the interview data through re-reading transcripts again and again. Reading the whole transcripts and highlighting all texts that appear to represent changes and challenges that parents faced in the transition to parenthood, or something they expressed stress and strain.
Stage3: Coding	The TSM served as the pre-specific creature for guiding analysts to identify meaningful text units and coding at the individual level. Any texts that could not be categorized by the code list would be given a new code. New code would be added to the code list.
Stage4: Developing general themes	Inducing similar codes to form sub-themes and themes, which is a fundamental step for dyadic analysis.
Stage5: Dyadic analysis	The couples were matched to conduct dyadic analysis at this stage. This process entailed examining the similarities and differences within the data of each dyad. Dyadic codes were identified from the comparison, focusing on capturing the collective experiences and perspectives of the couples rather than individual experiences.
Stage6: Developing dyadic themes	Dyadic themes were created based on the dyadic codes reflecting couples’ transition experience rather than only individual experience.
Stage7: Refining the themes	Two analysts discussed their analysis results together including the codes’ contents and the definition of each theme and sub-theme. If they could not agree, all the co-authors would discuss together until a consensus was reached.

**Table 3:** The example of analytical units, codes, sub-theme and theme.

Analytical units	Codes	Sub-theme	Theme
*F2: “As a father, I cannot feel the baby moving like the child’s mother does. If the baby is not moving at all, I feel even less of his real presence. So every time I finish work and come home, the first thing I want to do is to see my wife, and think about what my little one has been up to in her belly today.”*(roles)	Doubting and feeling the changes brought by role transition.	Doubt and feeling	Role integration
**M12: “I got heavy hypertensives gravid that vomited over 10 times in 1 day. I never thought my pregnancy would be harder than everyone ease’s. It was only after my symptoms subsided that my husband and I regained the joy of becoming parents.*”*(roles and responsibilities)	The role of self-awareness in the process of accepting the new role.	Resistance and identification	
*M14: “If it were not for my parents wanting a child, I would definitely choose to go with the flow and not endure so much pain to undergo IVF. But now that my belly is so big and the baby is about to come, I feel like the bond between me and the baby is getting stronger during this process (pregnancy). I am also looking forward to his arrival.”* (roles and relationships)	The process of reconciling self-perception with others’ expectations regarding the parental role.	Conflict and reconcillantion	
*M6: “The process of waiting for fetal examination results can be excruciating. Even if the previous examination results were normal, there is still a lot of worry with each new one.”*	Worrying about the child’s growth and development, whether it’s progressing normally and if they are healthy.	Health concerns about children	Health risk
*F18: “In the early pregnancy of my wife, she often did not feel well. So, I had to take her to emergence for examinations and treatments. My workload was intensive in the daytime, and I could not rest at night. I felt very fatigued at that time. […] I am very worried about my health status.”*	The impact of role transition on one’s own health condition.	Parental health challenges	
*F15: “Even washing the baby model is so difficult. I feel especially nervous every time I attend the dad’s class. Sometimes my wife jokes around, saying that I might drown the baby like this.”* (knowledge)	The knowledge and skills that mothers and fathers should acquire.	Learning of knowledge and skills	Dilemma of preparation
*F9: “Whenever she has a prenatal check-up, I always accompany her. So, I have to take leave from work in advance and reserve time for the prenatal check-up day.”* (responsibilities)	The shift in priorities in personal time allocation after pregnancy.	Redistribution of time	
*F4: “House lawn plays the most of our salaries. The payment for examination and nutrition is a big deal of money. It is impossible for us to save money.”*	The increase in financial expenses after having children.	Aggravated financial stress	
*M13: “After I got pregnant, my husband did all chores. I often get strongly helpful support from him. He wants me to have a relaxed pregnant experience. I am very grateful to him and have more confidence during pregnancy.”* (responsibilities)	The division of household tasks among family members changes after pregnancy.	Adjustment of responsibility allocation	
*M3: “It’s been raining every day this week, and my parents will not let me go out at all. They say it’s too slippery outside and it’s not safe. I cannot go anywhere except the balcony every day.”* (roles，relationships, and responsibilities)	Restricting movement and activities to stay away from all potential dangers.	Restriction on mobility and activity	Protective isolation
*F13: “All my friends are aware of my wife’s pregnancy, and as a result, they refrain from inviting me to engage in late-night activities. They want to avoid any potential conflicts or disagreements between my wife and me that may arise from such social engagements. “* (relationships)	A decrease in social life after becoming parents.	Being socially shunned	
*M9: “Last time when I felt the baby kicking me, I asked my husband to share this fantastic moment to feel the fetal movement. It seemed he wasn’t as excited as me. I wonder if he does not care about the baby and me. I felt very hurt for being ignored. I hope my husband could show more love to me.” /F9: “I have no idea why I have to be so excited. I mean the existence of fetal movement represents our baby is healthy and normal. If there is no fetal movement, I will definitely be in tension because our baby is abnormal. Her unstable emotions and doubts sometimes hurt me. I do not want her to feel unhappy or lonely, so I will try my best to take good care of her.”* (responsibilities and relationships)	The desire for more attention as a mother. /The responsibility of fathers to meet the desire of mothers.	Extra-care requirement	Multi-dimensional expectations
*M16: “Our cultivation plans for son or daughter are different. For my son, we decide to make money to buy houses and cars for his future marriage. For our daughter, we will invest more in cultivating her talents like singing, dancing, or playing the instruments.”* (roles and responsibilities)	Ideas or fantasies about what the future child will be like.	Ideal child fantasy	

## 3 Results

Five themes and thirteen sub-themes emerged from the interviews representing five kinds of parental transition shock.

### 3.1 Role integration

When mothers and fathers integrated parental roles into their self-concept, they initially perceived the changes in many aspects brought by role transition. They claimed they were experiencing self-concept disorganization and re-stabilization.

Both mothers and fathers indicated integrating their parental role was a fluctuational process with complex emotions toward parental role. When they experienced emotional ups and downs, they felt these emotions stimulated them to proactively perceive, learn, clarify, and accept the obligations and expectations of the parental role.

#### 3.1.1 Doubt and feeling

Upon discovering the pregnancy, parents exhibited heightened attentiveness to the daily changes occurring in the fetus. This heightened awareness was particularly evident in couples in the early stages of pregnancy who relied on medical examinations to confirm the presence of the fetus. During this period, it was common for parents to experience doubts regarding the reality of the pregnancy and make continuous efforts to perceive the existence of the fetus. Couples with a history of miscarriage exhibited a notable low confidence regarding the smooth progression of their current pregnancy and the possible of becoming parents. Consequently, they were more susceptible to expressing doubts and uncertainties during this period.

*F4: "We once lost a baby in the last pregnancy. I was afraid I hold too much hope to have a healthy baby […] until the mid-pregnancy, the maternity examinations showed that the baby was healthy. I really think I’m going to be a father."*

Unapparent physical changes and pregnancy reactions also contributed to the doubts experienced by some parents. Several mothers expressed confusion over the relatively mild pregnancy reactions they were experiencing in the early stages, which differed from their expectations. Additionally, fathers tended to have longer-lasting doubts and feelings of uncertainty compared to mothers. Since they could not directly perceive the existence of the fetus, they relied on examination reports and conversations with their wives for confirmation.

*M11: "I didn’t feel any different in the early pregnancy. So, I often pay more attention to my body change to insure I am really pregnant. Sometimes, I even engaged in vigorous exercise without realizing my pregnancy."*

*F14: "If it wasn't for the pictures of the ultrasound, I am hard to believe (becoming a father)."*

#### 3.1.2 Resistance and identification

Self-concept refers to an individual’s perception of themselves. The majority of mothers and fathers in the study mentioned that the presence of a fetus prompted them to reassess their self-concept, as it involved taking on the responsibilities of parenthood and meeting societal expectations associated with it. This transition resulted in a sudden detachment from their familiar self-concept. Many participants expressed resistance towards parenthood because they felt that their current self-concept was in-congruent with the parental role. In particular, parents who experienced unintended pregnancies exhibited greater resistance to the parental role due to a lack of prior preparation.

*M1: "This baby came at a very bad time, I used to have an opportunity for promotion this year. The unplanned pregnancy wastes my efforts in the past 3 years. Under the pressure from my family, I have to continue (the pregnancy)."*

Over time, participants in the study reported a gradual re-establishment of feelings of control and familiarity, allowing them to adapt to the changes associated with transition and achieve role identification. They expressed a growing ability to think and act from a parental perspective, and to experience the range of emotions typically associated with the parental role.

*M12: "I got heavy hypertensives gravid that vomited over 10 times in one day. I never thought my pregnancy would be harder than everyone ease's. It was only after my symptoms subsided that my husband and I regained the joy of becoming parents."*

#### 3.1.3 Conflict and reconciliation

In Chinese culture, the child carries the hopes and aspirations of multiple family generations, and family members have high expectations of parents. Many participants in the study mentioned experiencing arguments and quarrels with family members who demanded that they behave “like a parent” according to their expectations. Parents expressed feeling trapped by these external expectations of the parental role imposed upon them by their family members.

*M14: "My mother-in-law doesn’t allow me to use stretch mark oil. She blamed me and said that ‘You are a bad mother’. I am a mother, but I am also myself."*

*F4: "My wife said a qualified father shouldn’t waste time on enjoyment, instead of making money for our family. I think she is being unreasonable."*

On the other hand, the conflicts with others also served as a catalyst for parents to recognize and understand the role expectations associated with parenthood, prompting them to engage in self-adjustment. Through repeated communication with their families and personal introspection, parents were able to reconcile their own expectations with those of others regarding the parental role. This process facilitated their adaptation and acceptance of the role expectations placed upon them.

*F1: "I hold a traditional belief that a responsible father should not let his wife work too hard. Despite this, my wife continued to work after becoming pregnant. I initially wanted her to quit her job and stay at home to focus on nurturing the fetus. However, she emphasized her passion for her job and believed that working hard could set a positive example for our baby. After having a thorough discussion, we have managed to find a compromise where I now accompany her to and from work every day."*

### 3.2 Health risk

The significant physical and mental challenges experienced by parents during this transition period often led to concerns and worries about the health status of both the fetus and themselves. Parents found themselves caught in a cycle of persistent anxiety, constantly worrying about potential health issues that could arise.

#### 3.2.1 Health concerns about children

Given China’s previous family planning policy, which encouraged many families to have only one child, the level of concern among parents for their fetus was particularly high. All participants expressed intense anxiety while awaiting the results of antenatal check-ups. Some parents mentioned being highly attuned to fetal movements, any physiological discomfort experienced, and even superstitious signs associated with Chinese traditional culture that they considered “unlucky.”

*M2: "I haven’t felt her movement for 2 days. So, I come here (Obstetrics outpatient) mainly to confirm my baby is OK."*

*M3: "My right eye has spammed many times in the past 3 days (representing bad luck in Chinese culture), I am afraid something bad may happen to my baby."*

#### 3.2.2 Parental health challenges

By comparing the interview records of fathers and mothers, we found that both fathers and mothers experience psychological and physiological hardships. Mothers reported experiencing physical discomforts because of pregnancy, such as vomiting, sleep issues, edema, and pain due to the pressure of the enlarged uterus. These physiological challenges often led to physical discomfort and emotional distress. Some mothers expressed feeling mentally vulnerable and uncertain about how to regulate their emotions. Fathers commonly experience anxiety regarding the health of both the fetus and the mother during pregnancy. Similarly, mothers often worry about the development of the fetus and their own health. It was a quite common worry among new parents. However, during the analysis process, one surprising result emerged: fathers expressed concerns about their own health. This unexpected finding was attributed to their experience of a decline in well-being while providing intensive care for their wives day and night.

*M4: "It is very difficult for me to have a good sleep. I usually wake up at night, because my huge belly is weighing me down. Pregnancy reaction and lack of sleep make me very irritable."*

*F18: "In the early pregnancy of my wife, she often didn’t feel well. So, I had to take her to emergence for examinations and treatments. My workload was intensive in the daytime, and I couldn’t rest at night. I felt very fatigued at that time. […] I am very worried about my health status."*

### 3.3 Dilemma of preparation

In parents’ efforts to cope with the challenges of transition, all participants actively engaged in preparation. However, parents reported encountering various difficulties during the preparation process, which left them feeling disoriented, upset, and fatigued.

#### 3.3.1 Learning of knowledge and skill

Both mothers and fathers expressed their commitment to learning medical knowledge and acquiring care-giving skills in preparation for their new roles as parents. However, due to their limited direct experience, they reported feeling stressed about the complexity of medical knowledge and the lack of hands-on practice opportunities.

*F6: "My wife and I decide to attend the new parents class to learn some knowledge and skill about babysitting. I can still remember that I got in a flurry when the teacher taught us how to do newborn bathing. Even if we were washing a baby model, I am still afraid that I would hurt it."*

Another aspect raised by some participants was the lack of reliable learning sources. Many expectant parents mentioned relying on internet searches to find information related to pregnancy. However, they had concerns about the accuracy and credibility of the information found online. Some parents suggested that hospitals should offer regular maternity training and lectures to provide reliable and up-to-date information to expectant parents.

*F7: "Although the internet is developed that we can get many information today. It is still difficult to identify useful and reliable information."*

*M5: "Sometimes we find some bad news about pregnancy on TikTok, no matter true or not, we will be anxious and upset about the fetus’s health status."*

One father emphasized that it is not the mother who should learn, but rather the father who should prioritize learning baby care-giving skills.

*F7: "Honestly, mothers need a lot of rest after giving birth. During that time, it is the father's responsibility to take care of the baby. However, men are naturally not as skilled in care-giving, so we need more professional education and practice."*

#### 3.3.2 Redistribution of time

The time required for pregnancy-related activities, such as antenatal examinations and training, also posed a significant time burden for parents. Participants expressed stress and concern about managing their time effectively and making appropriate time plans. Many participants mentioned the steps they took to prepare for antenatal examinations, such as scheduling appointments in advance, requesting permission for work absences, and waiting at the hospital.

*M2: "I always have appointments with a senior obstetric doctor who has many regular pregnant women. It is hard to reserve his appointment and I have to wait for a long time until my turn. I am very anxious during waiting time."*

*F8: "In order to have a smooth transition to being a skilled father, I attend a new father class every Saturday. It used to be my entertainment time that I could play e-games to relax. After my wife get pregnant, I change my enjoyable weekend plan into a learning weekend plan. The time for entertainment has decreased a lot."*

#### 3.3.3 Aggravated financial stress

“Monthly expenses” emerged as a common concern among the majority of participants. They expressed worry about the increased costs associated with pregnancy, including medical expenses, nutritional supplements, and various preparations for the baby’s arrival. Furthermore, some mothers experienced a decrease in salary during pregnancy due to physical limitations and taking maternity leave, further exacerbating their stress and financial burden.

*M4: "I can only get a basic salary because of work absence […]. Even when I work, it's hard for me to finish the workload I used to do because of my physical strength, so my income has decreased a lot."*

During this period, fathers were primarily viewed as the main source of financial income, and they frequently mentioned experiencing “financial stress” more than mothers. However, fathers often chose not to express this stress to their wives, possibly to alleviate any additional burden or anxiety their partners might feel.

*F4: "House lawn plays the most of our salaries. the payment for examination and nutrition is a big deal of money. It is impossible for us to save money." (He frowned when mentioned the difficulties in the family's financial situation.)*

*F8: “I choose not to share my financial stress with my wife. I want her to be happy and free from any additional psychological burden.”*

A small number of participants expressed that the current policy support in terms of reducing fertility costs was insufficient. For instance, couples without local medical insurance mentioned their inability to receive immediate medical expense reductions.

*F5: "Our medical insurance was registered in our hometown. So, a part of the cost could not be reimbursed by medical insurance immediately in this province."*

#### 3.3.4 Adjustment of responsibility allocation

The increasing physical burden during pregnancy posed challenges for mothers in fulfilling their family duties. As a result, parents recognized the need to reassess the allocation of responsibilities to adapt to the situation. Fathers usually took on additional family responsibilities, including household tasks that were previously managed by mothers.

*F7: "We live in a special situation that pushes us to confront and navigate the changes on family responsibility allocation. It is true that the process can be difficult as I must do more for our family. However, we believe that this adjustment serves as evidence of our shared desire to improve our family and overcome the obstacles together."*

Through daily interactions, several couples gradually developed a mutual understanding and establish a suitable allocation of responsibilities based on their unique circumstances. When mothers and fathers supported and empathized with each other during the transition, their awareness and willingness to embrace the parental role were strengthened.

*M13: "After I got pregnant, my husband did all chores. I often get strongly helpful support from him. He wants me to have a relaxed pregnant experience. I am very grateful to him and have more confidence during pregnancy."*

### 3.4 Protective isolation

The lifestyle and social relationships of parents undergo significant changes during pregnancy. On one hand, parents proactively make changes to prioritize maternal health and safety. On the other hand, people in their social circle monitor and support them in maintaining a healthy lifestyle and protecting them from potential risks. However, this increased attention and care can sometimes make parents feel isolated from their previously unrestricted world.

#### 3.4.1 Restriction on mobility and activity

Some participants shared their experiences of being prohibited from engaging in activities that could potentially jeopardize maternal health and safety by family members, healthcare professionals, and friends. Parents expressed that they felt compelled to comply with these restrictions, which had an impact on various aspects of their lives, including mobility, dietary choices, sleep routines, exercise habits, and smoking behaviors. These restrictions made them feel a sense of loss of freedom and suppressed their natural instincts.

*M5: "These days are raining. Due to the wet and slippery ground, my parents don’t allow me to go out. I could only spend time on boring TV series. I was locked up all day like a prisoner!"*

*F17: “I was driven to the balcony by my mother-in-law, because second-hand smoke may harm my fetus. […] I did want to have a smoke sitting on the soft sofa.”*

#### 3.4.2 Being socially shunned

Both mothers and fathers acknowledged that their relationships with friends had changed since becoming parents. Many participants mentioned that they were no longer as actively invited to socialize and engage in recreational activities with their friends. Regardless of the specific reasons, parents commonly expressed feelings of loneliness and a sense of emptiness during this period. They also felt hesitant and shy to openly and directly discuss the reasons behind this change in their relationships. Some of them attributed this relationship change to their friends did not want to bother them in nourishing the fetus.

*M5: "Since I got pregnant, my friends have seldom asked me to hang out together. I know they just consider my health and safety, but I still feel ignored."*

*F13: “All my friends are aware of my wife’s pregnancy, and as a result, they refrain from inviting me to engage in late-night activities. They want to avoid any potential conflicts or disagreements between my wife and me that may arise from such social engagements.”*

While other participants considered that they felt embarrassed on social occasions when their friends proactively accommodated them. So, they said they chose to turn down friends’ invitations.

*M7: "When I came out with my colleagues and friends, they were very anxious and careful about my safety. I felt unease to be treated differently…I thought I was bothering them. So, I don’t want to have fun with them until childbirth."*

### 3.5 Multi-dimensional expectations

After becoming pregnant, many parents started dreaming about their future and envisioning their ideal parental life. These dreams serve as goals they strive to achieve. Through their imagination, they found joy and excitement about what lies ahead. However, it is important to note that when parents realize the disparity between their dreams and reality, they may experience feelings of frustration.

#### 3.5.1 Extra-care requirement

Through dyadic analysis, it was observed that mothers and fathers held different expectations and needs within this sub-theme. Mothers expressed a strong desire for increased attention and care during pregnancy from their husbands, families, and colleagues. They viewed their husbands as the most important source of support and attention during this transitional period. In cases where mothers felt that they were not receiving sufficient attention and care from their husbands, they expressed feelings of dissatisfaction, insecurity, and even anger.

*M9: "Last time when I felt the baby kicking me, I asked my husband to share this fantastic moment to feel the fetal movement. It seemed he wasn’t as excited as me. I wonder if he doesn’t care about the baby and me. I felt very hurt for being ignored. I hope my husband could show more love to me."*

On the other hand, fathers perceived themselves as responsible for ensuring the emotional well-being and stability of mothers, and they had high expectations of themselves in meeting their wives’ care needs. They experienced notable anxiety in trying to meet the expectations and demands placed upon them by mothers. When they perceived themselves as falling short in meeting these needs, they experienced feelings of guilt and inadequacy.

*F9: "I have no idea why I have to be so excited. I mean the existence of fetal movement represents our baby is healthy and normal. If there is no fetal movement, I will definitely be in tension because our baby is abnormal. Her unstable emotions and doubts sometimes hurt me. I don’t want her to feel unhappy or lonely, so I will try my best to take good care of her."*

#### 3.5.2 Ideal child fantasy

Dreaming about their future babies brought a sense of fulfillment to parents. They expressed that these fantasies guided their preparations, including choices related to clothing, toys, and even cultivation plans based on the anticipated gender of their babies. Parents found joy in envisioning their babies’ characteristics and personalities, and they believed that aligning their preparations with these dreams would create an ideal environment for their child’s development.

*M16: "Our cultivation plans for son or daughter are different. For my son, we decide to make money to buy houses and cars for his future marriage. For our daughter, we will invest more in cultivating her talents like singing, dancing, or playing the instruments."*

A few participants specifically mentioned the fantasy about their babies’ intelligence and future development. They hoped their children could be smart and successful in academic performance and in jobs.

*F13: "If our baby is clever, he/she will get great scores and have access to better job opportunities."*

*M8: “I hope he/she could be admitted into an elite university. Then, he/she could do an easy and high salary job not like us earn money so hard.”*

## 4 Discussion

This qualitative study provides valuable insights into the experiences of first-time parents during the transition to parenthood in mainland China. By employing the TSM, we were able to systematically investigate and analyze these experiences. Notably, this study goes beyond previous research by including a dyadic analysis that compares and contrasts the perspectives and experiences of both mothers and fathers (Grilo et al., 2018). This aspect has been relatively unexplored in previous qualitative studies, making our findings unique and significant. Surprisingly, our findings revealed minimal differences between mothers and fathers in their experiences of the transition to parenthood. This highlights the shared nature of the emotional journey and expectations that parents go through during this period. Our findings also shed light on the various challenges faced by parents, including physical discomfort, limited coping resources, and a lack of social connections. However, it is important to note that the sub-theme of extra-care requirements did highlight distinct expectations between the two partners. These findings contribute to a comprehensive understanding of transition to parenthood within the Chinese cultural context and serve as a valuable foundation for future investigations into gender differences in the transition process.

### 4.1 Internal adjustment: mixed emotions and expectations as a new parent

Most of the parents in this study, regardless of their gender, expressed a range of emotions when transitioning into parental roles, including both happiness and discomfort. This result is consistent with previous studies that have found that parents often experience anxiety and uncertainty when facing the arrival of a new life (Philpott et al., 2017; Bécotte et al., 2022). Unlike previous studies that primarily viewed negative emotions as risk factors for maternal health and emphasized the importance of preventing them (Fenwick et al., 2012; Staneva et al., 2017; Baldwin et al., 2018), our study suggests that certain negative emotions can actually act as catalysts for parents’ inner growth and role adaptation. For instance, most participants in this study experienced stress related to societal expectations placed on parents, particularly those coming from their families. However, they still made efforts to understand and incorporate their family’s expectations of parental roles into their self-concepts. This aligns with the Role Acquisition Theory, which proposes that individuals tend to conform to others’ expectations during social interactions (Schumacher, 1995). It implies that negative emotions may also have positive effects on parental role adaptation. Hence, instead of solely focusing on stringent prevention, healthcare providers should place greater emphasis on closely monitoring negative emotional responses, ensuring that they remain within a secure range.

Expectations refer to the beliefs, predictions, or assumptions that individuals hold about future life, outcomes, or behaviors, which play a crucial role in shaping people’s cognition, emotions, and behaviors (McCarthy et al., 2021). While there is a growing body of knowledge regarding mothers’ expectations, the understanding of fathers’ expectations was relatively limited (Fenwick et al., 2012). Our studies identified that parents’ expectations mainly revolved around care requirements and what children would be like. Through dyadic analysis, a difference between mothers and fathers in terms of their expectations was observed. While mothers expressed a desire to receive more care and feel loved, fathers had the expectation of being able to fulfill the care requirements of the mothers to the best of their abilities. This finding may be attributed to the stereotyped gender roles that often assign the role of nurturer to mothers and the role of support provider to fathers (Lévesque et al., 2020). Thus, fathers may feel stress about a greater responsibility to fulfill the care-giving expectations of mothers, which may influence their expectations during the transition to parenthood. It highlights the need for fathers to be aware of and responsive to the emotional needs of mothers (Figueiredo et al., 2018), while also emphasizing the importance of mothers openly expressing their expectations and concerns. Meanwhile, fostering a supportive and nurturing environment for both parents, can contribute to a positive transition to parenthood and enhance the well-being of the entire family (Figueiredo et al., 2018; Alves et al., 2020; Brandao et al., 2020).

Regarding their expectations for their children, the parents in this study emphasized their children’s future development, such as academic performance and having a good job. This finding is a notable cultural characteristic specific to China, as it contrasts with a Danish study that revealed Danish mothers placed a higher priority on the health and well-being of their children (Ladekarl et al., 2022). One possible explanation is that Chinese parents place a strong belief in the correlation between academic achievement and future development opportunities, particularly since China is a highly competitive environment. Additionally, traditional beliefs contribute to the notion that a child’s academic performance reflects the success of the parents, which may further reinforce the value placed on academic success in Chinese parenting.

### 4.2 External challenges: physical burden, lost preparation, and lack of social connection

The transition to parenthood presents challenges not only for mothers but also for fathers. While a significant amount of attention has traditionally been placed on the health status and needs of mothers, the experiences and needs of fathers have often been overlooked or given less attention (Furber et al., 2009; Renzaho and Oldroyd, 2014; Ladekarl et al., 2022). In our study, it was unexpected to find that four fathers reported an increasing physical burden experience during the transition period. These fathers mentioned that the demands of intensive care providing and household chores significantly increased their energy consumption and physical exertion, which were reported in a few studies conducted in the postnatal period (Martins, 2019; Lévesque et al., 2020), rather than the prenatal period. While the attention to the poor mental health condition of fathers is growing (Leach et al., 2016; Philpott et al., 2017; Martins, 2019), the exploration of the physical health concerns specific to fathers has been rarely seen. Further studies could explore the physical well-being of fathers during this critical period.

All participants in our study displayed positive attitudes towards preparation as an essential aspect of managing the journey of pregnancy and childbirth. They recognized the importance of being ready and actively sought out information, support, and resources to navigate this transition phase. However, despite their proactive approach towards preparation, we discovered a lack of accessible resources and clear instructions to assist them in their transition to parenthood. This dearth of preparation resources and guidance may impede their ability to adequately prepare for the demands and responsibilities of pregnancy and parenthood (Renzaho and Oldroyd, 2014; Erasmus et al., 2020). Chinese National Health Commission has asked hospitals to provide health education for pregnant couples for several years. Based on the interview results, it appears that participants had limited access to health education provided by hospitals. This can be attributed to two primary factors: limited availability of time to attend on-site education sessions and the absence of regular education programs (Backstrom et al., 2022a,b). With the maturation of digital technologies like virtual reality (VR) and augmented reality (AR), hospitals and healthcare providers have the opportunity to create online health education courses. By utilizing VR and AR, they can enhance the educational experience for parents (Backstrom et al., 2022a). This approach would increase the coverage of reliable health education and provide parents with convenient access to educational materials. They would have the flexibility to learn at their own pace and convenience, improving their preparation for pregnancy and childbirth.

Another significant dilemma of preparation is the aggrieved financial stress of parents in China. The cost of living and child-rearing in China is relatively high, which places a significant financial burden on parents and even has been considered to be the important reason why Chinese couples are less likely to have children (Ning et al., 2022; Yang et al., 2023). Moreover, the social and cultural context in China places an emphasis on providing the best possible care for children. This societal expectation, coupled with the desire to give their child a good start in life, places additional financial strain on parents. What’s worse, the fact is that household income is even reduced during pregnancy due to limited salaries for mothers due to unpaid leave or reduced work hours. It further exacerbates the financial burden and poses great challenges for parents as they navigate the transition to parenthood. Hence, more financial support projects for parents should be posted in China to alleviate the financial challenges faced by parents during the transition to parenthood. Strategies like reducing the taxes (on pregnancy and child raising), providing more job insurance for mothers and fathers during the transition period, and expanding social insurance programs to cover a wider range of expenses related to pregnancy and child-raising are highly recommended. These practices will not only alleviate the financial burden but also contribute to the overall well-being and quality of life for parents and their families, and even encourage the fertility intention (Yang et al., 2023).

Most of the participants in this study reported the reduced opportunity to socialize as frequently as they desired it or used to be before pregnancy, which resulted in feelings of isolation and neglect. These findings align with a study conducted in Canada, where participants reported similar experiences (Lévesque et al., 2020). In addition to the Canadian study, whose participants attributed the reduction of social connection to time constraints and energy decrease, the findings of this study shed light on another significant factor: the influence of Chinese family-centered culture (Chang et al., 2010; Lau, 2012). Parents in this study subtly revealed that cultural norms create a barrier between themselves and their friends, impacting their social connections. Chinese family-centered culture strongly emphasizes family values and the central role of parenthood. Consequently, parents were compelled to prioritize their maternity health and safety, decreasing the time and attention available for maintaining social connections with friends. The diminished social connections can have a harmful impact on maternity health and well-being, as it can contribute to negative pregnancy-specific experiences (Malik et al., 2023).

### 4.3 Strengthens and limitations

Our study had three strengths. (1) With the theory instruction, this study gets a more comprehensive understanding of Chinese parents’ first-time transition to parenthood. (2) Separate interviews can enhance the credibility and validity of the study’s findings by providing participants with a comfortable and confidential space to express their thoughts and feelings about their spouses. (3) The dyadic analysis method further deepens the understanding of the transition to parenthood on gender difference and relationship functioning. There were also two limitations of our studies. First, only a small number of people give birth unmarried in China. So, we did not include the population of unmarried parents. A stable marriage relationship may provide more sense of security to mothers and fathers. The unmarried parents may have different obstacles and perceptions towards the transition. Second, all of our participants reported different extents of transition shock after becoming parents. However, this qualitative study could not investigate the extent of transition shock. There is no specific measurement for parents’ transition shock, so an objective measurement should be developed considering the parental transition shock.

## 5 Conclusion

The findings of this study illuminate the formidable challenges encountered by fathers and mothers during the process of transition to parenthood within the distinctive cultural context of mainland China. Both parents are confronted with the expectation of swift adaptation to their parental roles, yet they often encounter obstacles in accessing reliable and beneficial coping resources. It is noteworthy that fathers bear the significant responsibilities of financial provision and care-giving for the mother, often without readily available avenues for seeking assistance. These insights underscore the importance of including fathers as a crucial support population during the transition period. Health providers and policymakers should collaborate to establish comprehensive support systems encompassing financial assistance, skills education, and the creation of a parent-friendly social environment.

## Data availability statement

The datasets presented in this article are not readily available due to ethical concerns about confidentiality. Requests to access the datasets should be directed to the corresponding author, TZ, tyzeng@tjh.tjmu.edu.cn.

## Ethics statement

The studies involving humans were approved by This study received ethical approval (No.TJ-IRB20220705) from the Ethics Committees of Tongji Hospital of Tongji medical college of Huazhong University of Science and Technology. The studies were conducted in accordance with the local legislation and institutional requirements. Written informed consent for participation in this study was provided by the participants’ legal guardians/next of kin.

## Author contributions

XL designed and implemented the study, conducted the data analyses, and wrote the manuscript. TZ and SN assisted with the design of the study and the data analyses and collaborated with the writing and editing of the manuscript. LJ and PQ collaborated with the design of the study, data analysis and editing of the manuscript. MW assisted with the design of study and data analysis. All authors contributed to the article and approved the submitted version.
